# Registration and segmentation of cine and late enhancement cardiac magnetic resonance images

**DOI:** 10.1186/1532-429X-11-S1-P76

**Published:** 2009-01-28

**Authors:** Gilion Hautvast, Cybele Ciofolo-Veit, Marcel Breeuwer

**Affiliations:** 1 Philips Healthcare, Best, Netherlands; 2 Philips Healthcare, Paris, France

**Keywords:** Cardiac Magnetic Resonance, Cardiac Magnetic Resonance Image, Late Enhancement, Ring Detection, Contour Propagation

## Introduction

Cardiac Magnetic Resonance (CMR) can be used to assess myocardial function and viability by acquiring cine and late enhancement (LE) images. Quantitative assessment of myocardial function and viability requires delineation of the myocardial contours in both images. To overcome the burden of manual delineation, automatic delineation methods for cine CMR images exist. However, automatic delineation of the myocardium in LE CMR images is more challenging due to the lack of features between blood and scar tissue and variations in inversion times and contrast injection delays. Therefore, we propose to register the cine and LE images such that the LE images can be automatically delineated by transforming the cine contours, which may have been obtained automatically. In this respect, recently published SCMR guidelines for cardiac magnetic resonance (CMR) imaging [1] drastically reduce the complexity of the registration problem by stating that late enhancement (LE) CMR images have to be acquired in the same views as cine CMR images.

## Purpose

The purpose of our work is to quantify the influence of different scanning procedures and cine CMR delineation methods on the accuracy of our automatic LE CMR delineation method.

## Methods

We have used cine and LE CMR images from 32 patients, acquired between 2004–2007. The cine scans consisted of 10–14 slices and 20–25 phases, whereas the LE scans consisted of 10–12 slices. All images were 256 × 256 and covered fields of view of 320–460 mm (cine) and 344–494 mm (LE). Three experts delineated all images. Golden standard contours were obtained by averaging contours. Inter-observer variability and contour accuracy were measured using Root-Mean-Square (RMS) positioning errors with respect to the golden standard.

The cine CMR images were delineated manually, semi-automatically and automatically. Semi-automatic delineation is performed using a contour propagation method. Automatic delineation is performed using a deformable template that is initialized using a ring detection procedure. The resulting contours were transformed to delineate the LE CMR images. The appropriate transformation was obtained by performing affine registration between cine and LE images that maximizes normalized mutual information in a coarse-to-fine approach using conjugate gradient optimization. Registration is performed between the LE slices and neighboring slices (to address through-plane motion) and phases (for accurate contour interpolation) in the cine scan. To anticipate on gross patient motion, initial translations are estimated by localizing the myocardium using a ring detection method.

## Results

**Figure 1:**
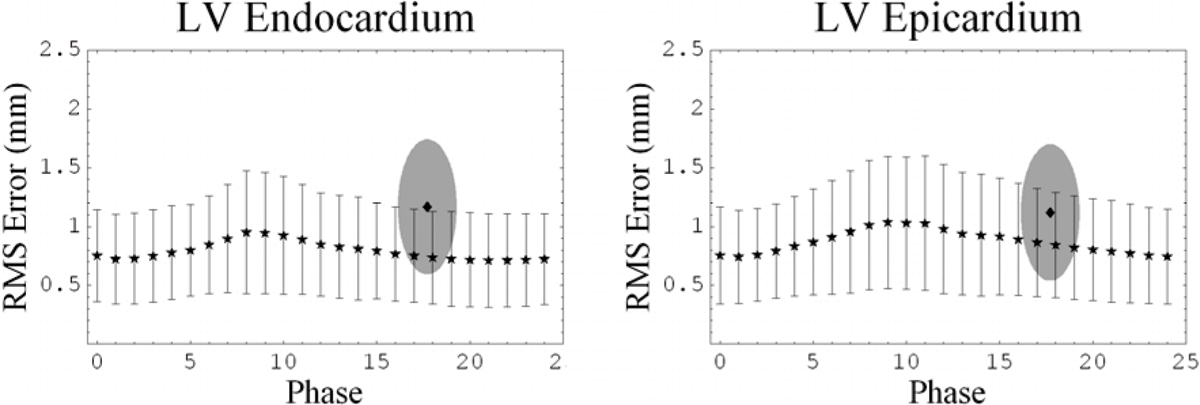
**Variability of manual LV contours**.

In cine CMR, the inter-observer variability over all phases was small, 0.80 ± 0.43 mm and 0.89 ± 0.48 mm for the endocardial and epicardial contours respectively, as compared to LE CMR, 1.17 ± 0.57 mm and 1.12 ± 0.58 mm (figure 1).

Our automatic segmentation method provided endocardial and epicardial contours at LE CMR with RMS errors of 2.20 ± 1.15 mm and 2.08 ± 1.02 mm respectively given manual contours at cine CMR images. At the equivalent phase of the cine CMR images, contour propagation resulted in endocardial and epicardial contours with RMS errors of 0.32 ± 0.42 mm and 0.19 ± 0.29 mm respectively, whereas for automatic detection RMS errors of 1.87 ± 2.35 mm and 1.64 ± 2.17 mm were obtained. Table 1 shows the resulting RMS positioning errors after transforming cine contours, (either manually drawn, propagated or automatically detected) at all cases and divided between cases acquired according the recent SCMR guidelines (6/32), at identical positions (aligned) and older cases (non-aligned).

**Table 1:** RMS positioning errors in mm for the LV endocardial and epicardial contours

	Manual	Propagated	Automatic
**LV Endocardium**			
All cases	2.20 ± 1.15	2.23 ± 1.15	2.54 ± 1.38
Aligned cases	1.25 ± 0.65	1.42 ± 0.72	1.97 ± 0.83
Non-aligned cases	2.34 ± 1.15	2.37 ± 1.16	2.64 ± 1.43
**LV Epicardium**			
All cases	2.08 ± 1.02	2.15 ± 1.06	2.53 ± 1.11
Aligned cases	1.41 ± 0.35	1.49 ± 0.53	1.72 ± 0.68
Non-aligned cases	2.18 ± 1.05	2.26 ± 1.09	2.76 ± 1.11

## Conclusion

We developed a new method for segmenting LE CMR images given delineated cine CMR images. Our new method provided very accurate contours for LE CMR images acquired according the recent SCMR guidelines [1], while maintaining reasonable accuracy in older, more difficult cases. Furthermore, the method is less sensitive to errors in initial contours if the images are acquired according recent guidelines.

## References

[1] Kramer CM (2008). J Cardiovasc Magn Reson.

